# Awake Blue Laser Excision and Balloon Dilation of Oropharyngeal Stenosis in High‐Risk Patients

**DOI:** 10.1002/ohn.70363

**Published:** 2026-08-17

**Authors:** Abbey L. Landini, Cyrus W. Abrahamson, Andrew P. Stein

**Affiliations:** ^1^ Northwestern University Feinberg School of Medicine Chicago Illinois USA; ^2^ Department of Otolaryngology–Head and Neck Surgery Northwestern University Chicago Illinois USA

**Keywords:** awake airway surgery, difficult airway, head and neck cancer, laser surgery, oropharyngeal stenosis

## Abstract

Oropharyngeal stenosis is a rare condition that can develop following head and neck cancer treatment, leading to progressive dyspnea, dysphagia, and dysphonia. Patients with symptomatic dyspnea may benefit from endoscopic surgery. However, head and neck cancer survivors can have severe trismus, limited neck extension, and altered laryngeal landmarks, which make rigid endoscopic surgery under general anesthesia high‐risk or potentially not feasible without a tracheostomy tube. We describe an awake approach for the management of oropharyngeal stenosis for 2 head and neck cancer survivors with high‐risk airways. Briefly, under monitored anesthesia care in the operating room, a flexible channeled nasolaryngoscope was utilized to perform awake blue light laser excision of stenosis followed by balloon dilation. Postoperatively, both patients reported symptomatic improvement, and objective increases in airway patency were noted at clinic follow‐up. This operative technique represents a feasible, well‐tolerated approach for high‐risk airway patients with oropharyngeal stenosis.

Oropharyngeal stenosis represents a rare condition that can arise following treatment for head and neck cancer, leading to dysphagia, dyspnea, and dysphonia.^1^ For patients with progressive dyspnea, management aims to improve airway patency while optimizing voice and swallowing. Conservative treatment includes endoscopic laser excision, balloon dilation, and antifibroblastic agents.^1^ For severe cases, open surgery can expand the oropharynx utilizing local or free tissue transfer.^2^

Laryngeal surgery under general anesthesia can be risky in head and neck cancer patients, especially if they have trismus, poor neck extension, and/or altered laryngeal landmarks, making rigid endoscopic exposure and intubation challenging or impossible.^3^ In such cases, awake procedures under monitored anesthesia care (MAC) are an alternative. Limited reports describe the awake treatment of laryngotracheal stenosis with balloon dilation ± laser excision, including Jiam et al's study showing awake CO_2_ laser surgery improves dyspnea with low intra‐operative conversion to general anesthesia.^4^, ^5^ However, these studies focus on the glottic/subglottic airway, and no reports exist describing awake treatment of oropharyngeal stenosis.

This article highlights the safety and utility of blue light laser excision of stenosis followed by balloon dilation for treatment of oropharyngeal stenosis in patients with high‐risk airways. This technique was utilized for 2 patients who developed oropharyngeal stenosis with progressive dyspnea following radiation for head and neck cancer. This study was deemed exempt by the Northwestern University Institutional Review Board (STU00223568).

## Case Presentations

### Patient Histories

Patient 1 is an 80‐year‐old male with a history of stage III/IV HPV‐positive right tonsillar squamous cell carcinoma treated with chemoradiation 18 years prior. He remained gastrostomy‐tube dependent after treatment and developed significant neck fibrosis, severe trismus, and mandibular osteoradionecrosis requiring open reduction/fixation. Comorbidities included aspiration pneumonia, thoracic aortic aneurysm, and coronary artery disease. He presented with dyspnea worsening over 6 months. Flexible laryngoscopy revealed circumferential scarring at the level of the epiglottis, leading to ~50% airway obstruction (Figure 1A).

**Figure 1:**
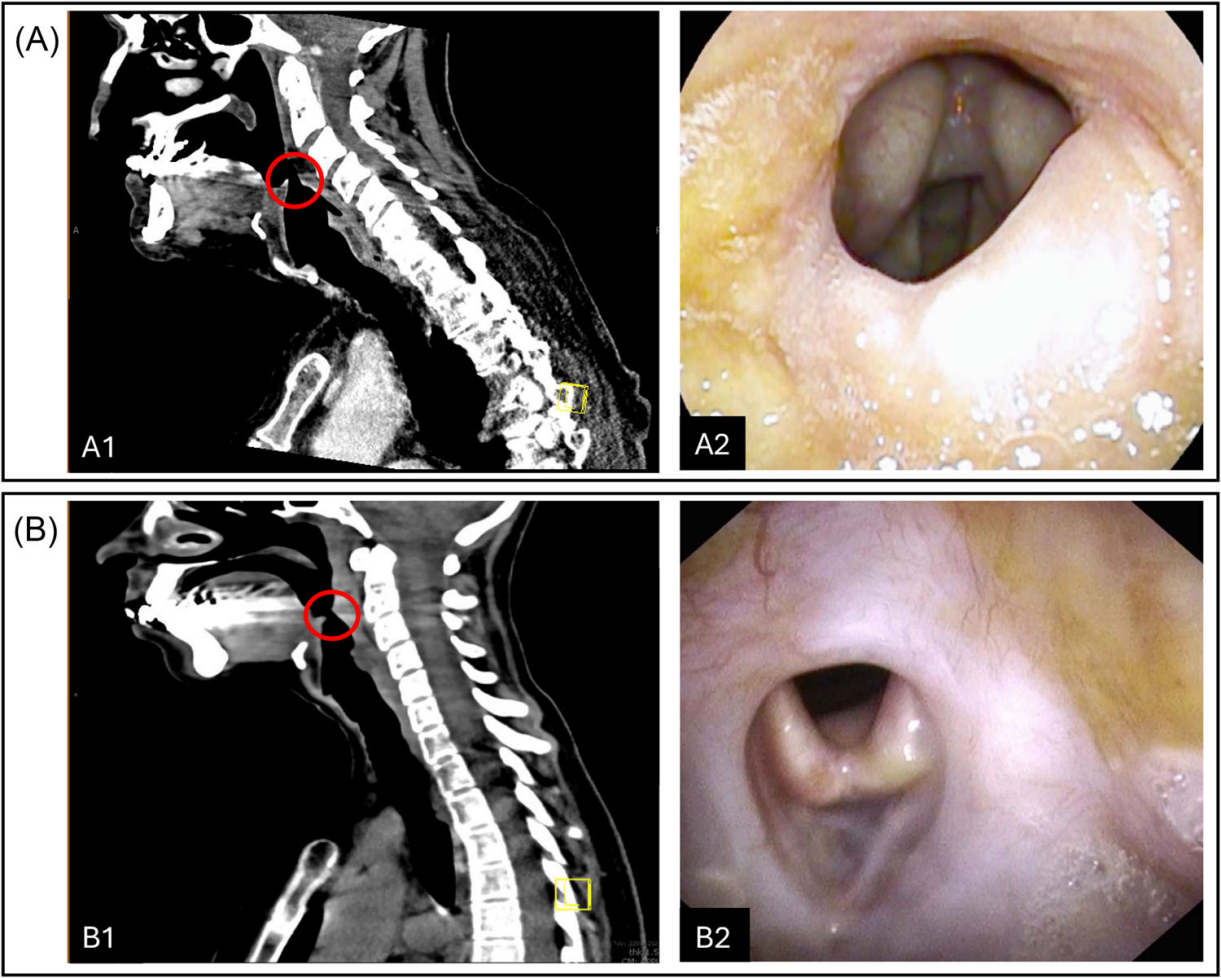
Preoperative imaging and endoscopic findings for Patients 1 and 2. (A) Sagittal computed tomography (CT) scan of the neck for Patient 1 demonstrates oropharyngeal narrowing around the epiglottis (red circle; A1), which was confirmed on flexible nasolaryngoscopy (A2). (B) Preoperative sagittal CT scan of the neck for Patient 2 reveals oropharyngeal stenosis (red circle; B1), and flexible nasolaryngoscopy shows approximately 70% narrowing of the oropharynx centered around the tip of the epiglottis (B2).

Patient 2 is a 32‐year‐old female with a history of nasopharyngeal carcinoma treated with chemoradiation 15 years prior complicated by upper esophageal stenosis requiring frequent dilations and gastrostomy‐tube dependence. She presented with progressive dyspnea over 1 to 2 years and also had severe trismus, limited neck extension, and prior difficult intubation. Laryngoscopy showed circumferential oropharyngeal stenosis around the tip of the epiglottis narrowing the airway ~70% (Figure 1B).

### Endoscopic Technique

Procedures were performed under MAC in the operating room. The anesthesia team titrated medications (typically midazolam 1 mg/mL and/or dexmedetomidine 4 mcg/mL in 0.9% NaCl) to balance spontaneous ventilation with sedation and comfort. Operative steps included (**Video** **1**):

1. Anesthetize nasal cavities with topical 4% lidocaine and oxymetazoline.
2. Nebulize 5 milliliters of 4% lidocaine.
3. Introduce flexible channeled nasolaryngoscope transnasally. Evaluate oropharynx, larynx, and stenosis. Additional 2% lidocaine delivered to oropharynx, supraglottis, and glottis.
4. Thread blue light laser fiber through scope. Perform laser radial excisions throughout oropharyngeal stenosis, alternating nares to optimize access. Laser settings were typically 7.5 to 10 W, 40 milliseconds ds pulse duration, and 300 milliseconds pulse pause. The laser could be used to address any oozing/bleeding during the surgery. Fortunately, neither patient developed bleeding that required a specific intervention during the procedure.
5. Inject triamcinolone 40 mg/mL into excision sites using a sclerotherapy needle (25‐gauge, 100 cm Interject Injection Therapy Needle Catheter; Boston Scientific) via scope.
6. Advance a guidewire (0.038 in × 260 cm Jagwire Straight Tip High Performance Guidewire, Boston Scientific) through scope and into trachea. Remove scope while maintaining wire.
7. Pass a balloon dilator (CRE Pulmonary Balloon Dilation Catheter, 12‐13.5‐15 mm or 15‐16.5‐18 mm, Boston Scientific) over wire to span stenosis. Balloon/wire in one nostril with scope in other.
8. Balloon dilation with patient‐coordinated breath holding.
9. Remove balloon/wire. Assess airway.

**Video 1:** Awake blue light laser excision and balloon dilation of oropharyngeal stenosis. Intraoperative video demonstrating awake transnasal flexible endoscopic blue light laser excision of oropharyngeal stenosis followed by guidewire‐assisted balloon dilation. Patient maintained under monitored anesthesia care with spontaneous ventilation throughout the procedure.

### Back‐Up Equipment

Since the patients remained awake under MAC, a tracheostomy tray was not kept on standby in the room. However, our anesthesia team maintained back‐up airway equipment nearby, including a GlideScope, fiberoptic bronchoscopes, and multiple endotracheal tube sizes in case conversion to general anesthesia and nasotracheal intubation were required. Direct laryngoscopy equipment was also readily available outside the OR if rigid laryngoscopy became necessary.

### Patient Outcomes

Patient 1 reported improved breathing and mild voice improvement. Laryngoscopy showed significant improvement in his oropharyngeal airway at initial postoperative visit (Figure 2A). Mild restenosis was observed at 4.5 months; however, no additional procedures were required through 1 year postsurgery.

**Figure 2:**
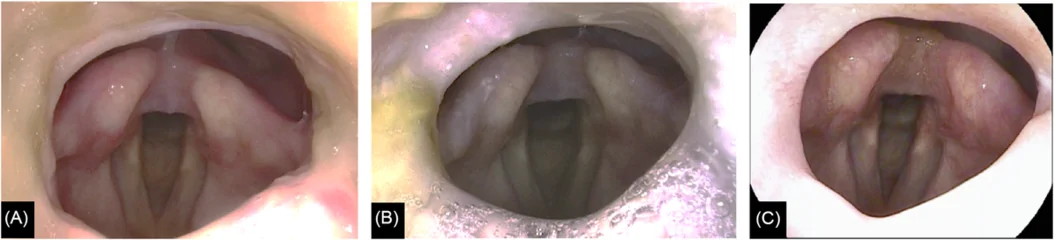
Postoperative clinic endoscopy for Patient 1. Flexible nasolaryngoscopy shows great improvement in airway caliber 9 days after surgery (A). Airway patency remains improved at 6 weeks (B) and shows mild restenosis 4.5 months postsurgery (C).

Patient 2 had marked improvement in breathing and swallowing function. Laryngoscopy confirmed improved patency initially, but airway caliber gradually decreased over time (Figure 3). She underwent this procedure 2 more times with similar benefit. Her second procedure occurred 11 months after the initial intervention, and the third surgery was 10 months later as a prophylactic measure to optimize her airway during pregnancy.

**Figure 3:**
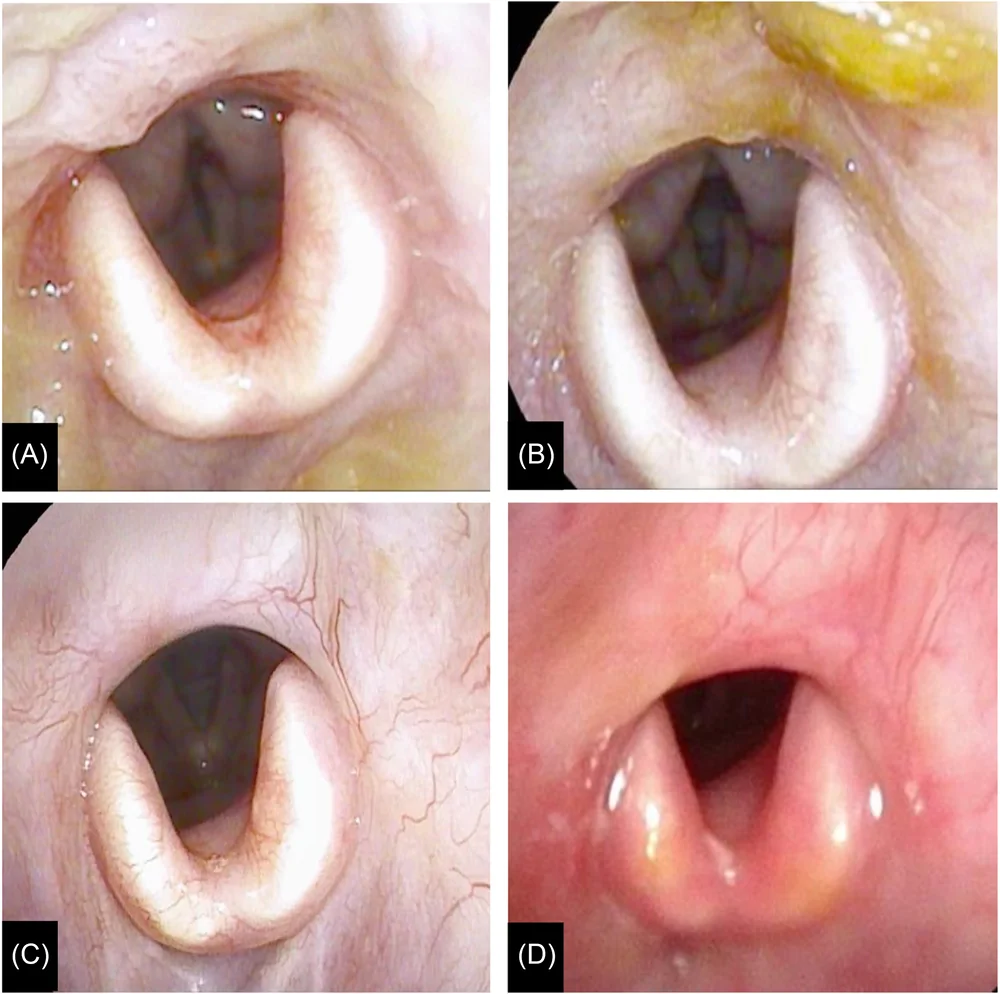
Images from Patient 2's postoperative flexible nasolaryngoscopies. Endoscopy 2 weeks postoperatively shows nice improvement in oropharyngeal airway caliber (A). Mild narrowing demonstrated 7 weeks postsurgery (B). Restenosis gradually increased over time as depicted 5.5 (C) and 8.5 months following surgery (D).

## Discussion

Awake laser excision with balloon dilation offers a promising option for managing oropharyngeal stenosis in high‐risk airway patients. This technique eliminates the need for general anesthesia with endotracheal intubation and rigid laryngoscopic exposure, which may not be feasible for patients with trismus, poor neck extension, and/or altered airway anatomy.

Both patients tolerated the procedure well and achieved improvement in their dyspnea and airway patency. As with all airway interventions, restenosis remains a challenge. Patient 2 required a repeat procedure 11 months after the initial intervention. Recurrence can occur along this timeline even with more typical endoscopic modalities to treat airway stenosis, indicating awake blue laser excision of stenosis with balloon dilation offers durable relief comparable to traditional approaches.^1^ Moreover, for patients needing repeat interventions, the awake approach provides a safer technique in those who would otherwise have limited options or require a tracheostomy tube to undergo surgery requiring general anesthesia.

This study has limitations, most notably the small cohort. We hope to evaluate this technique in a larger group moving forward. However, demonstrating the safety and feasibility of this approach adds valuable insight to the current literature surrounding the management of oropharyngeal stenosis in patients with complex airway anatomy.

## Conclusion

Awake blue light laser excision of stenosis with balloon dilation is feasible and well‐tolerated for select patients with oropharyngeal stenosis who are not optimal candidates for general anesthesia and/or rigid endoscopic exposure. This technique offers a safe alternative for restoring airway patency while avoiding tracheotomy and the risks of intubation. Further investigation is warranted to define this procedure's role within the broader landscape of airway stenosis management.

## Author Contributions

**Abbey L. Landini**, data curation, visualization, writing—original draft; **Cyrus W. Abrahamson**, data curation, writing—original draft; **Andrew P. Stein**, conceptualization, project administration, supervision, writing—review and editing.

## Disclosures

Meeting Information: This work was presented as an oral presentation at the Triological Society Combined Sections Annual Meeting, January 22‐24, 2026 in Orlando, FL.

### Competing interests

None.

### Funding source

None.

## References

[1] Herrington HC , Weber SM , Andersen PE . Modern management of laryngotracheal stenosis. Laryngoscope. 2006;116(9):1553‐1557. 10.1097/01.mlg.0000228006.21941.12 16954977

[2] Tompkins JJ , Vaughn CA , Shaikh FA , Stocks RM , Thompson JW . Palatopharyngoplasty with bilateral buccal mucosal graft repair to alleviate oropharyngeal stenosis. Int J Pediatr Otorhinolaryngol. 2015;79(9):1579‐1581. 10.1016/j.ijporl.2015.06.022 26145205

[3] Atkins JH , Mirza N . Anesthetic considerations and surgical caveats for awake airway surgery. Anesthesiol Clin. 2010;28(3):555‐575. 10.1016/j.anclin.2010.07.013 20850085

[4] D'Oto AD , Marshall CR , Ma Y , Young VN , Rosen CA . Outcomes in awake laser laryngeal stenosis surgery (ALLSS): a 5‐year retrospective review. Laryngoscope. 2025;2:803‐809. 10.1002/lary.70100 40923567

[5] Jiam NT , Dwyer CD , Rosen CA . Awake laser laryngeal stenosis surgery. Laryngoscope. 2021;131(5):E1633‐E1637. 10.1002/lary.29295 33264421

